# ANINet: a deep neural network for skull ancestry estimation

**DOI:** 10.1186/s12859-021-04444-6

**Published:** 2021-11-11

**Authors:** Lin Pengyue, Xia Siyuan, Jiang Yi, Yang Wen, Liu Xiaoning, Geng Guohua, Wang Shixiong

**Affiliations:** College of Information Science and Technology, Northwest University, Xi’an, China

**Keywords:** 3D skull models, Ancestry classification, Depth projection, ANINet, Cross-validation

## Abstract

**Background:**

Ancestry estimation of skulls is under a wide range of applications in forensic science, anthropology, and facial reconstruction. This study aims to avoid defects in traditional skull ancestry estimation methods, such as time-consuming and labor-intensive manual calibration of feature points, and subjective results.

**Results:**

This paper uses the skull depth image as input, based on AlexNet, introduces the Wide module and SE-block to improve the network, designs and proposes ANINet, and realizes the ancestry classification. Such a unified model architecture of ANINet overcomes the subjectivity of manually calibrating feature points, of which the accuracy and efficiency are improved. We use depth projection to obtain the local depth image and the global depth image of the skull, take the skull depth image as the object, use global, local, and local + global methods respectively to experiment on the 95 cases of Han skull and 110 cases of Uyghur skull data sets, and perform cross-validation. The experimental results show that the accuracies of the three methods for skull ancestry estimation reached 98.21%, 98.04% and 99.03%, respectively. Compared with the classic networks AlexNet, Vgg-16, GoogLenet, ResNet-50, DenseNet-121, and SqueezeNet, the network proposed in this paper has the advantages of high accuracy and small parameters; compared with state-of-the-art methods, the method in this paper has a higher learning rate and better ability to estimate.

**Conclusions:**

In summary, skull depth images have an excellent performance in estimation, and ANINet is an effective approach for skull ancestry estimation.

## Introduction

In the field of forensics, when identifying unknown corpses, forensic experts generally rely on experience to directly determine or use the sex chromatin in the tissue cells of the corpse or hormones in the blood to infer the sex, age, ancestry and other information of the deceased. Among them, ancestry estimation is one of the most important research contents in identification. In 2017, Murphy and Garvin [1] conducted research on human ancestry and gender, and the skull was proposed as a new biological feature and attracted increasingly attention of researchers in the field of forensics and archaeology. Compared with other bones of the human body, the skull can better reflect the differences between ancestries, and the skull is the most commonly used bone when estimating ancestry [2–9]. Some morphological characteristics of the skull are used to assess ancestry, such as cranial index [10], bone shape [11–13], facial protrusion [14], nasal bone shape [15], etc. When evaluating these unmeasurable characteristics, the interaction between gender and ancestry must also be taken into account. Research has shown that gender differences between different ancestors are different [16, 17]. Therefore, the determination of the unknown skull ancestry is the first and important step in the identification of forensic anthropology.

Traditional skull ancestry estimation methods are divided into morphological estimation methods and morphometric methods. The morphological estimation method refers to forensic experts observing the morphology of the skull and then making subjective judgments on the regions or features with racial differences in the skull to determine the ethnicity of the skull. This method is subjective and not highly appraisal. Because traditional morphological estimation methods completely rely on expert experience, the development of morphometrics provides new ideas and methods for ancestry estimation. Giles and Eillot [2] selected 8 measurement indicators of the skull to establish a discriminant to classify black and white Americans with an average accuracy of 85.0%. Hefner [3] used several statistical methods suitable for morphological characteristics, including logistic regression, Bayesian and k-nearest neighbors, and estimated skulls in Africa, Asia, Europe, and America. The accuracy of the classification was 84–93%, it is concluded that feature combination is better than individual feature combination. Osteoware [18] proposed 16 macromorphological features, which can be used to score standardized data collection, and can also be used to estimate ancestry within a statistical framework. The main purpose of this study is to test the utility of these characteristics in assessing ancestry. In 2012, Shao et al. [10] and others measured the craniofacial bones of 100 Guangxi Zhuang and 100 local Han adults and found that there are certain differences. In 2014, Klales and Kenyhercz [11] used the method proposed by Hefner and Osteoware to conduct experiments on black and white Americans, and the accuracy rate for ethnic classification was 86.6%. Wei et al. [12] used 10 characteristics of the brain to infer the discriminant equations of the yellow race and the white race. The blind test results of 16 samples were randomly selected, and the accuracy rate was 81.3% to 100%. Compared with traditional morphological estimation methods, morphological measurement methods only partially rely on expert experience, but require tedious measurement operations and has certain limitations.

With the development of computer technology, operators tried to introduce computer technology into the research process of skull ancestry estimation. Computer-aided measurement methods have replaced traditional manual measurement methods. The new method reduces the tedious measurement process and improves measurement accuracy. In 2016, Mikoláš et al. [13] used photogrammetry and laser scanning to digitally process the skull and used FIDENTIS Analyst software to calculate the difference metric between grids to quantify the 3D skull shape. Using discriminant function analysis and canonical variation analysis for further processing, the gender and ancestry accuracy rates of 80 Brazilian skull samples of different ethnicities were 82.5% and 63%, respectively. In addition, scholars have also devised an automated computerized skull estimation method that uses cranial side view, back view, and top view (from 3D scans) combined with elliptical Fourier analysis (EFA) to estimate the ancestry and gender of black and white American samples. The experiment result indicated that the ethnic classification accuracy rate of the side view reached 92.4%, which was about 22% and 13% higher than the rear view and the top view, respectively. Since the statistical methods used for skull population trends did not provide a photorealistic representation, Jodi et al. [19] used modern computer graphics methods to quantitatively analyze the skull photo data and generated photorealistic and objective skull shape samples after mathematical deduction and calculation, which provided new ideas for the study of ancestry and gender estimation in forensic anthropology.

To sum up, the methods of ancestry estimation have made great progress at home and abroad, but these methods generally have shortcomings such as strong subjectivity, need to manually label feature points, and consume manpower and material resources. In addition, Holliday and Falsetti [20] showed that there are differences in physical characteristics among American yellow people, African yellow people, and Asian yellow people, hence Asian people cannot directly apply the discriminant equation established abroad when identifying skeletal ancestry. Therefore, the research based on the automatic gender and ancestry estimation of the three-dimensional skull has a wide range of scientific research and practical value in forensic, anthropology, facial restoration, and other cutting-edge research fields. This paper proposed two skull projection methods, combined with ANINet to realize the automatic estimation of skull ancestors.

The main contributions of this paper are summarized as follows:

- We have established the first complete Mongolian multiethnic craniofacial depth image data set. It has two subdata sets: a local depth image set and a global depth image set, which provide data support for automated skull biometric research.
- We propose ANINet, a new neural network for skull ancestry estimation, which has high recognition accuracy and few parameters.

The second section of this paper describes the data preprocessing work; the third section describes the research ideas, projection methods, and network design; the fourth section verifies the recognition effect and effectiveness of the proposed method through experiments; finally summarize the full text.

## Experimental data

To complete our research, 95 samples of Asian population (55 males and 45 females) and 110 samples of Europeans (55 males and 55 females) were selected from the original CT data in Xi’an, China. All selected data are caseless data. All volunteers who participated in the collection were voluntarily collected, and they are between the ages of 20 and 60 years old.

### Skull reconstruction and denoising

To eliminate the influence of the internal structure of the skull and other impurities, we select the outer boundary of the skull from the CT image, perform a three-dimensional reconstruction, and denoise the data to obtain the three-dimensional model data of the skull.

#### Skull reconstruction

This paper uses the moving cube algorithm, Matching Cube algorithm (MC algorithm) to reconstruct skull CT data. The MC algorithm [21] is a classical algorithm for reconstructing regular discrete three-dimensional spatial data fields from computed tomography images. One cube is constructed from two adjacent skull CT images. Every four vertices on the cube are composed of four pixels on the same CT image, and then each layer is processed in turn from left to right, front to back, and top to bottom. In a cube, the gray value of the vertices of the cube is compared with the threshold value of the isosurface to obtain the edge that intersects the isosurface. The coordinates of the intersection point will be calculated for all intersection edges, that is, the coordinates of the intersection point of the cube and the isosurface. In this way, we get the coordinates of the triangle vertices on the isosurface, and finally calculate their normal vectors, superimpose the lighting effects, and generate a three-dimensional model.

#### Skull denoising

**Fig. 1:**
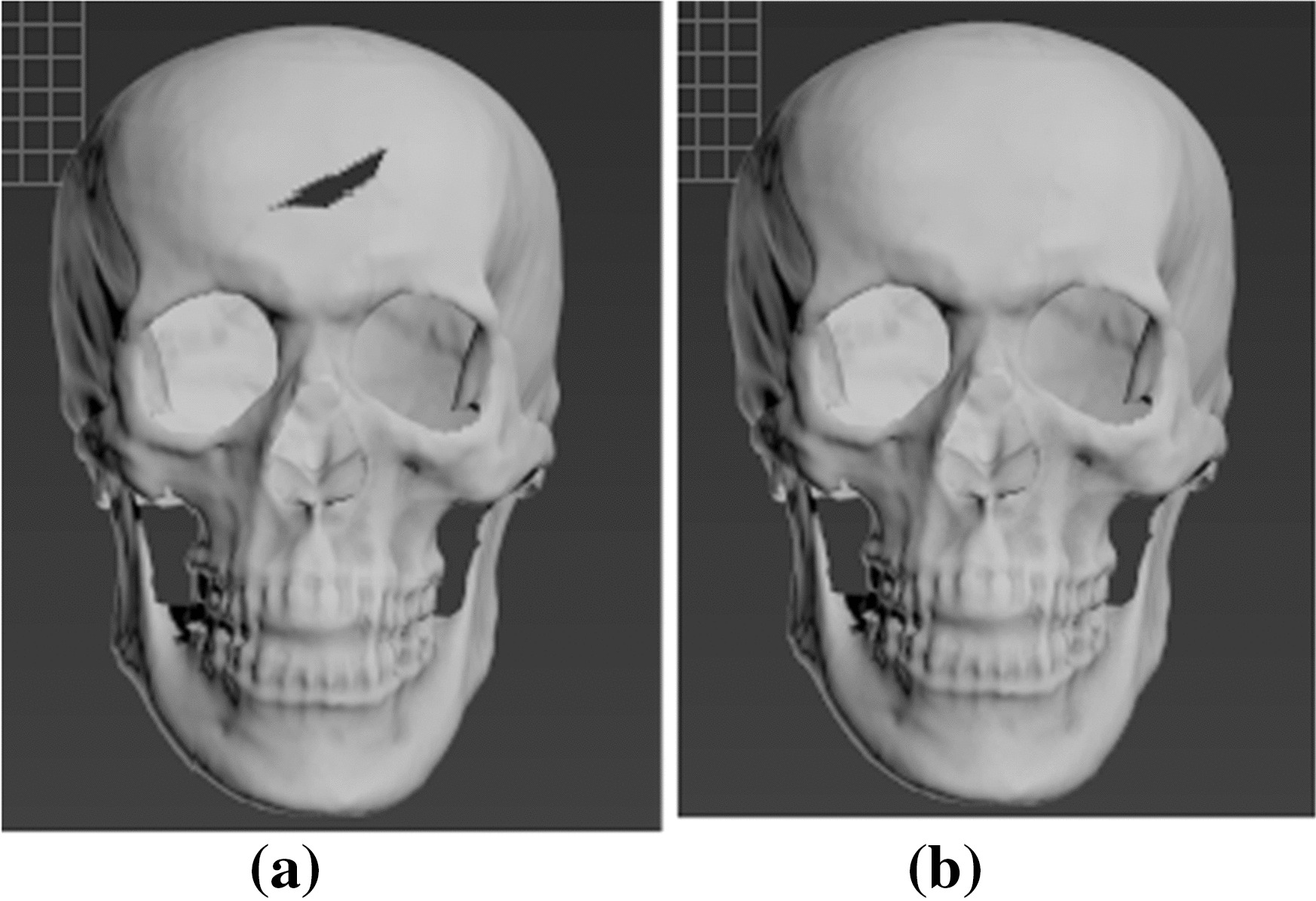
**a** 3D skull model with holes; **b** three-dimensional skull model after hole repair

This paper mainly performs de-redundancy and hole repair on the 3D skull model. It is executed using the reverse engineering software Geomagic Studio 2012. The specific steps are: (a) Open the Geomagic Studio 2012 software and import the reconstructed 3D skull model (.obj format); (b) Select the crop button under the polygon function to complete the de-redundancy operation; (c) Select the surface, plane, or fill the hole by means of a bridge. Figure 1 shows the effect of skull repair.

### Skull normalization

**Fig. 2:**
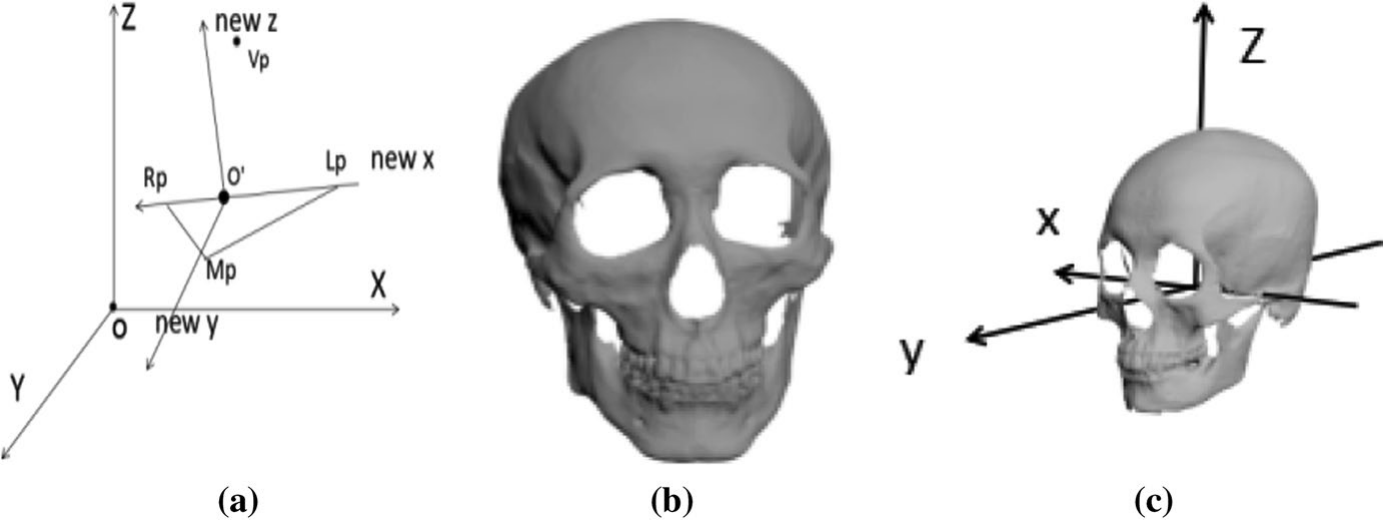
**a** Frankfurt coordinates system; **b** visualization of the 3D model of the skull; **c** skull in Frankfurt coordinates system

To eliminate the inconsistency of sample size and location, we adjust all data to a uniform scale and adjust to the Frankfurt coordinate system. The coordinate system is composed of four upper skull marks, which are the upper point Lp of the left ear door, the upper point Rp of the right ear door, the lower edge point of the left orbit Mp, and the point between the eyebrows Vp. The Frankfurt coordinate system is shown in Fig. 2a.

The intersection of the plane passing through the point Vp and the straight line formed by Lp, Rp is taken as the new origin o'; the line connecting the left and right ear gate points Lp, Rp as the x-axis; the straight line perpendicular to the plane formed by Lp Rp Vp and passing through the point o'is taken as z-axis; the y-axis is a straight line that is perpendicular to x and perpendicular to z. The 3D model of the skull is shown in Fig. 2b and the Frankfurt coordinate system of the skull is shown in Fig. 2c.

## Methods

**Fig. 3:**
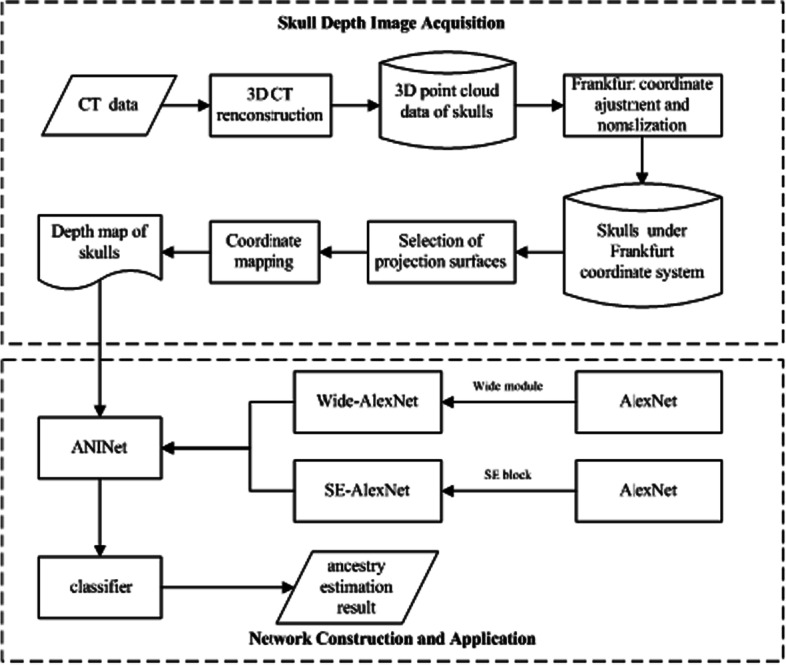
The framework of our method

This paper proposes a skull ancestry estimation method based on ANINet. The method mainly includes two processes: skull depth image acquisition and ANINet design. In the image acquisition stage, the three-dimensional skull is subjected to local and global depth projection to obtain the skull depth image, and then the skull depth image is smoothed, the image size is set to 160 × 160, and as the network input, the image is normalized to from 0 to 1; in the network design stage, the core of the network is the feature extraction process. We improve AlexNet [22], design and propose ANINet. First, introduce the wide module for feature extraction, and then combine the SE block [17] with the wide module to further learn the extracted feature channels to obtain the dependency between feature channels, strengthen important features, and suppress irrelevant features. Figure 3 shows us the framework of our method.

We confirm that all experimental protocols were approved by Northwestern University Ethics Committee and all methods were carried out in accordance with relevant guidelines and regulations. This study has informed consent which was obtained from all subjects.

### Depth projection

#### Local depth projection

**Fig. 4:**
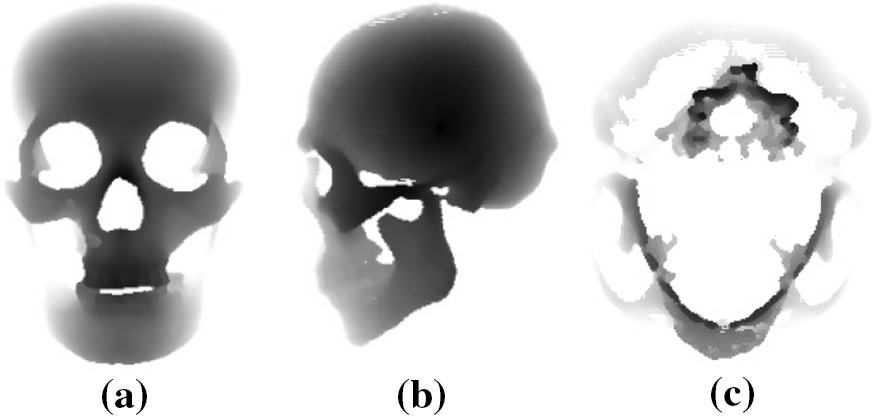
Projection renderings: **a** the front; **b** the left side; **c** the bottom

We project the front, left side, and bottom of the skull in Frankfurt coordinates system to produce projection images of three views. It is necessary to establish three projection surfaces firstly on the front, side, and bottom of the three-dimensional skull. The projection surfaces are, respectively, placed at tangent positions with the front, side, and bottom surfaces of the three-dimensional skull to ensure that the acquired image features were least lost. The projection surface sizes are set to 400 × 400 to ensure that the received skull depth images information were complete. We obtain the depth image of the three views finally by vertically projecting the 3D point cloud data onto the corresponding projection surface, and the distance from the point to the projection surface is taken as the gray value of this pixel point. The obtained deep images of the three views are smoothed. Projection rendering of the skull model as shown in Fig. 4, where Fig. 4a–c are the depth image of the front, left side, and bottom projections of the skull, respectively.

#### Global depth projection

**Fig. 5:**
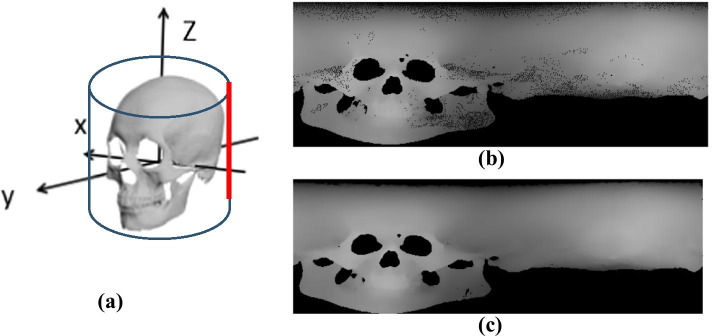
**a** The starting position of the projection surface of the skull; **b** Global depth projection; **c** Smoothed projection
- Image generation. Vertically projecting a point A from the point cloud data to the cylindrical c. Image generation. Vertically projecting a point A from the point cloud data to the cylindrical projection surface, intersecting at a point P(ρcosθ, ρsinθ, z), and the distance from the point to A to P is L, which is the gray value of the pixel point (i, j). We obtained and smoothed the global depth image of the skull by scanning all point clouds. Projection rendering as shown in Fig. 5, where Fig. 5b represents the depth image after global projection, and Fig. 5c represents the depth image after smoothing.

We take a global projection method to directly obtain a two-dimensional image containing the global information of the skull. The generation process is divided into three steps:

- Create a cylindrical projection surface. The cylindrical projection surface is built around the skull. The central axis of the cylinder is placed on the z-axis of the Frankfurt coordinate system. Since all skull models are normalized, the bottom radius of the cylinder is set at ρ = 1.3 and the height is fixed at h = 3. The starting position of the projection is the intersection line (Fig. 5a, red line), where the plane located xoz(x < 0) intersected with the cylinder.
- Coordinate mapping. To facilitate subsequent processing, the result of the projection has to be converted into the form of a picture. Assuming that the height of the generated image is H and the width is W, the pixel position (i, j) relationship between the projection surface and the blank image is established in the polar coordinates of the cylinder, as showed in Eq. (1). The polar coordinate equation of a cylinder is shown in Eq. (2).
  1 $$\left\{ \begin{array}{ll} {\text{i}} = \left[ {\left( {z + \frac{{\text{h}}}{2}} \right) \times \frac{H}{{\text{h}}}} \right] \hfill \\ j = \left[ {\theta \times \frac{W}{360}} \right] \hfill \\ \end{array} \right.$$
  2 $$\left\{ \begin{array}{l} {\text{x}} = \rho \cos \theta \hfill \\ y = \rho \sin \theta \hfill \\ z = z \hfill \\ \end{array} \right.$$

### ANINet (improved)

#### AlexNet

Convolutional neural networks mainly build multilayer networks and use higher-level features to represent low-level features. Through feature screening, the most robust and distinguishable features are obtained, and finally the features are used for classification or regression. Compared with CNN, AlexNet [22] deepens the network structure on the basis of it. It uses more convolutional layers and a larger parameter space to fit the data set and can learn richer and higher-dimensional images. Features and better classification results, which makes AlexNet the core algorithm model for image classification tasks. The AlexNet network model is divided into eight layers, including 5 convolutional layers and 3 fully connected layers. Each convolutional layer contains the activation function RELU, local response normalization (LRN) processing, and downsampling (pool processing). The main functions of the three fully connected layers in the network are different: the input of the first fully connected layer is the features extracted by the convolutional pooling layer, the second fully connected layer is mainly used for feature representation, and the last fully connected layer is mainly used for the classification of the classifier.

AlexNet obtains the loss of the true value and the predicted value through the forward propagation of the signal. The gradient descent method is used to minimize the loss function to adjust the weight parameters in the network layer by layer, and the accuracy of the network is improved through multiple iterations of training. The network has 650,000 neurons and 60 million learning parameters. At the same time, AlexNet is trained on two GPUs at the same time.

The skull ancestry estimation problem studied in this paper is a classification problem, and AlexNet is a very effective model to solve the classification problem. Therefore, based on the AlexNet model, this paper designs a network model that effectively solves the problem of skull ancestry estimation.

#### The wide module

**Fig. 6:**
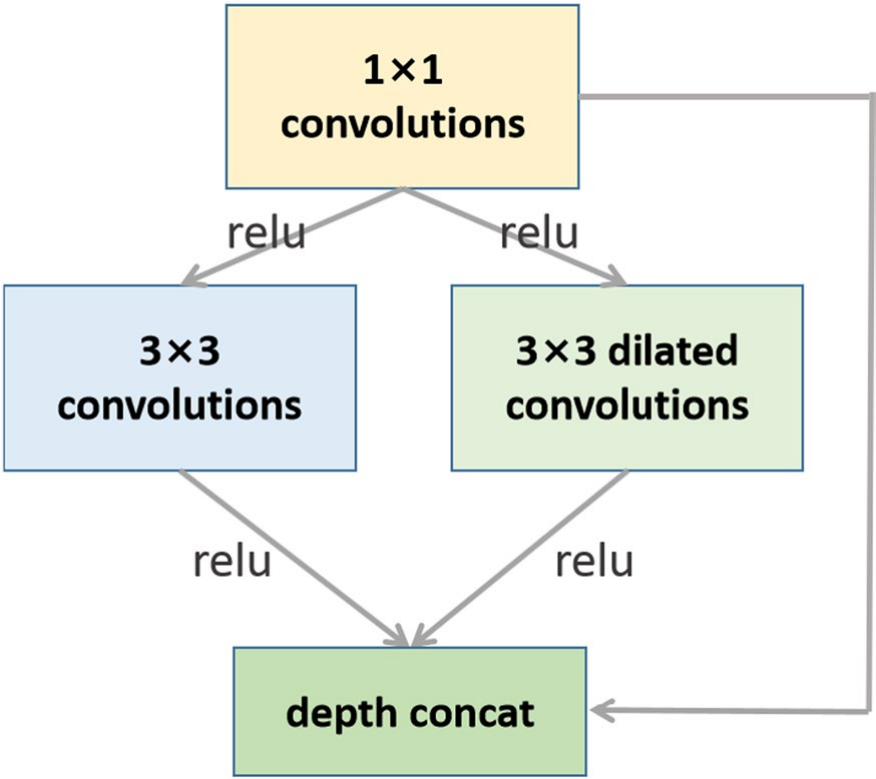
Basic structure of the wide module

Figure 6 shows the basic structure of a wide module. The output result of the upper layer is passed through 1 × 1 convolution kernels, and then connected to the different convolution kernels in the hybrid convolution layer, which contains normal convolution kernel of 3 × 3 size and dilated convolution kernels with a dilation rate k. Finally, the obtained features are channel-merged with the previous 1 × 1 feature layer. The 1 × 1 convolution kernels are mainly used for compression of feature channels and increasing nonlinear factors in the network. The 3 × 3 dilated convolutions can expand the receptive field without increasing parameters. The dilated convolutions can discover the relationship of nonadjacent features in the image, and it is not affected by the convolutions of the previous layer. In addition, this multi-channel merging method uses the principle of sparse matrix decomposition into dense matrix calculation to accelerate the convergence speed of the network, and it is not easy to make it over-fitting.

#### SE block

Squeeze-and-activation Network (referred to as SENet) [17] is a network structure proposed by Hu Jie's team. SENet won the ImageNet 2017 competition. The core idea of SENet is to train the model through the network to learn the weight of the feature channel according to the loss, so that the effective feature map has a large weight, and the invalid or small effect feature map has a small weight to achieve better results. The SE block is embedded in some original classification networks, which inevitably increases some parameters and calculations, but the effect is still acceptable. The core of SENet is the Squeeze-and-activation (SE) block. It is not a complete network structure, but a substructure composed of squeeze and activation, which can be embedded in other classification or detection models.

#### ANINet

AlexNet has made great breakthroughs in the field of image classification. Based on the advantages of convolutional neural networks, we converted the 3D point cloud data of skulls into images containing depth information, and then combined them with CNN to classify. In this study, the depth projection combined with the ANINet (ANcestry Identification Network) proposed in this paper is the first applied to the problem of skull ancestry estimation.

**Fig. 7:**
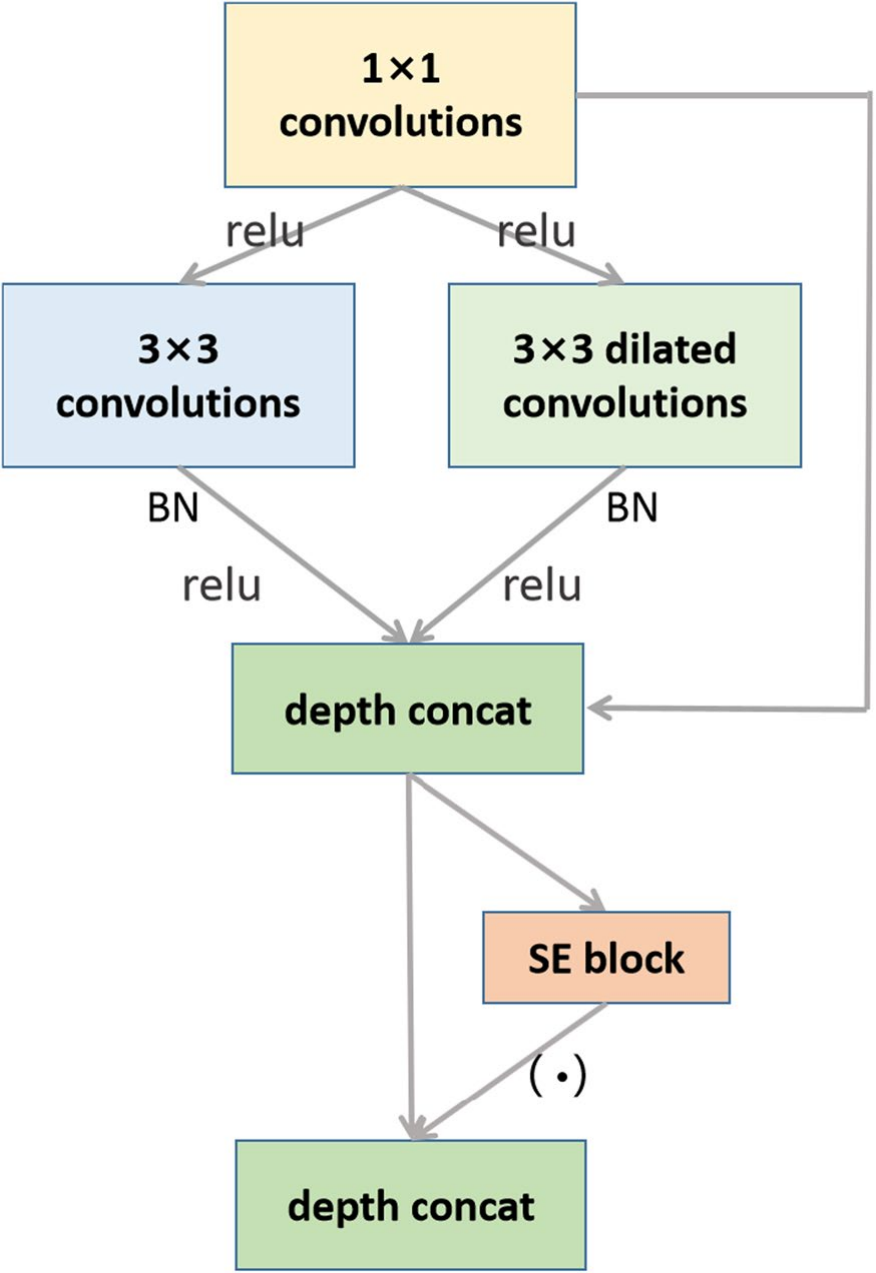
The SE-wide module structure

The core part of the network is the feature extraction part. We adjust the Wide module in “Local depth projection” section as the feature extraction part of the network, combining the SE block and the Wide module to further learn the feature channels extracted by the Wide module, obtain the dependency relationship of the feature channels, strengthen the important features, and suppress the features with little effect on the current task. The structure of the SE-Wide module is shown in Fig. 7.

Figure 7 shows the SE-Wide module structure. The feature extraction part of the network uses the SE-Wide module, the size of the convolution kernel is 3 × 3, and the hole convolution rate k is set to 2. The SE-Wide module combines the advantages of the SE block and wide modules to make the features more abundant and representative. The pooling layer of the network adopts a maximum pooling of 2 × 2, the activation function uses RELU, the output layer activation function is sigmoid, and the optimizer uses Adam. Besides, BN technology is used in the network to optimize data to prevent overfitting and accelerate convergence.

**Fig. 8:**
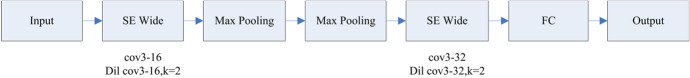
Network structure for ancestor recognition

We use the SE-Wide module to transform the AlexNet network and design the ANINet ancestry estimation network. The network structure is shown in Fig. 8. The input size of the network is 160 × 160. However, since the image acquired by the local depth method is 400 × 400 × 3, the acquired image needs to be scaled to 160 × 160 × 3 before training. The image obtained by using the global depth method is 629 × 301 × 1. Before training, an anisotropic scaling method is required. The long side of the image is scaled to 160, and the short side is scaled proportionally. Then the missing part is supplemented to obtain a 160 × 160 × 1 size image. When the two projection methods are combined for estimation, the three images obtained by local depth projection and one image obtained by global depth projection are scaled and then combined on the feature channel to obtain an input of 160 × 160 × 4.

## Experiments

**Fig. 9:**
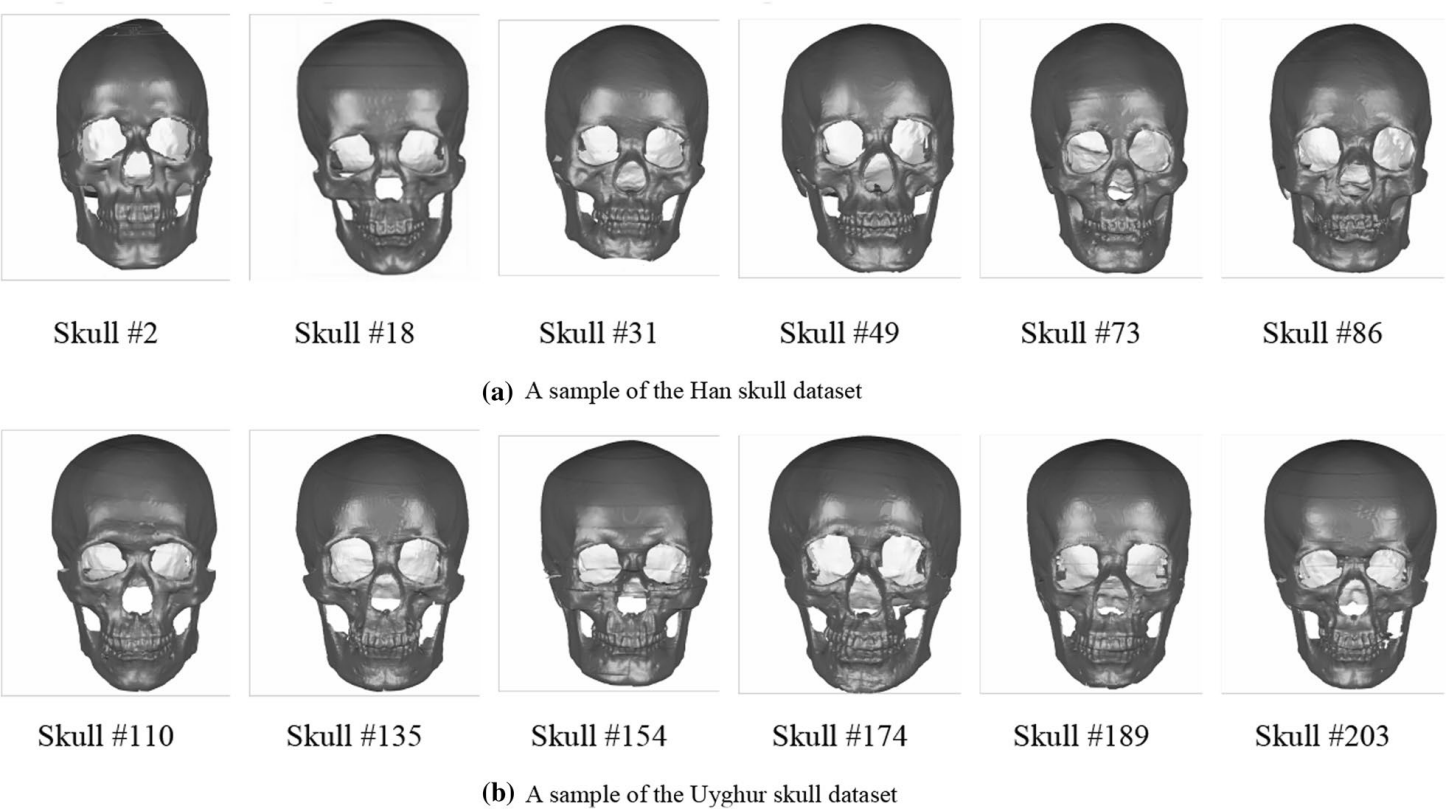
Visualization of experimental data

Before network training, we first divided the data set and expand the data. The data expansion step was completed during the acquisition of the depth projection. The three-dimensional model of the skull was slightly rotated and adjusted several times, and then the local depth projection and the global depth projection were used to obtain the image. Finally, we completed the work of ANINet training, parameter tuning, and so on. A sample of the dataset is shown in Fig. 9.

### Experimental results of local projection

**Fig. 10:**
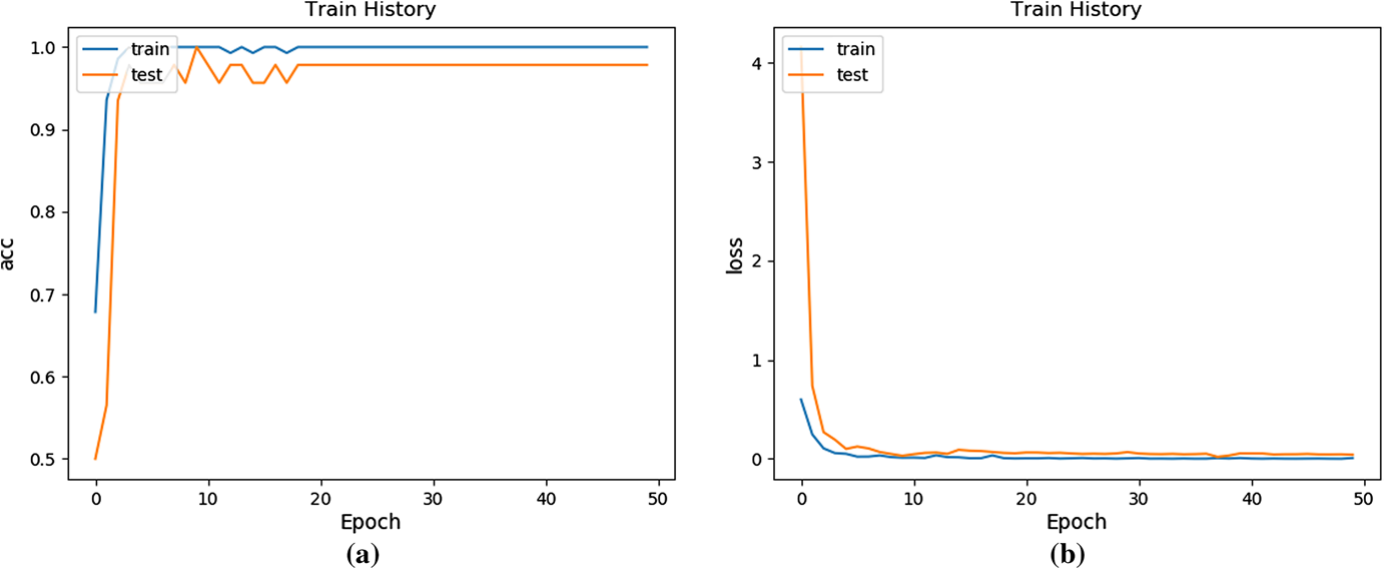
The accuracy and loss of using ANINet change with the number of iterations: **a** The curve of accuracy change; **b** The curve of loss value changer

This paper used the partial projection method to obtain 205 × 3 (including front view, left view and bottom view) depth images of 400 × 400 size, set the image size to 160 × 160 uniformly. The three depth images were merged by convolution and were input to the network. In the local projection mode, this experiment used fivefold cross-validation [23] to verify and adjusts the network model by changing the adjustable parameters in the network during the network training process, and obtains better results. Table 1 shows the network-related parameter settings, and Fig. 10 shows the curve of the accuracy and loss value with the number of iterations.

**Table 1:** The relevant parameter settings of the network

	Convolution 1	Convolution 2	Hidden	Output
Activation Function	relu	relu	relu	Sigmoid
Kernel/units	32	64	300	1
Optimizer	Adam			
Loss function	Logarithmic loss function

It can be seen from Fig. 10a that on the training set, the ANINet identification accuracy rate can reach 99.03%, and the ANINet identification accuracy rate on the test set can reach 98.21%. In the early stage of training, the accuracy of both increases as the number of network training increases. When the number of iteration steps reaches 20, the accuracy of the network for skull race identification reaches the highest value and tends to be stable. As can be seen from Fig. 10b, on the training set, the lowest ANINet loss value is 0.15, and the lowest ANINet loss value on the test set is 0.20. In the initial training stage, both loss values increase with the number of network training. However, when the number of iteration steps reaches 20 times, the loss value of the network reaches the lowest value and tends to be stable.

The ancestry classification studied in this paper is a binary classification problem, and the performance measure of binary classification will be used by researchers to measure the generalization ability of the binary classification model. The performance measurement evaluates the model's results based on the algorithm, the data, and the task requirements, which evaluates the quality of the model. In order to obtain accurate and comprehensive binarization classification performance indicators, we first needed to divide the sample into TP, TN, FP, and FN according to the combination of the true category and the model predicted category. TP means that the classification result is true positive, TN means true negative, FP means false positive, and FN means false negative. TP (Ture Positives) refers to the number of positive samples that are correctly predicted as positive samples, FP (False Positives) refers to the number of negative samples that are incorrectly predicted as positive samples, and TN (True Negatives) refers to Is the number of negative samples that are correctly predicted as negative samples. FN (False Negatives) refers to the number of positive samples that are incorrectly predicted as negative samples. And TP + TN + FP + FN = n, where n is the sample size. Binarized classification performance index can better measure the classification effect of the model, including recall, precision, accuracy, specificity and F1-score. It is defined as follows:

3 $${\text{Recall}} = \frac{TP}{{TP + FN}}$$

4 $${\text{Precision}} = \frac{TP}{{TP + FP}}$$

5 $${\text{Specificity}} = \frac{TN}{{TN + FP}}$$

6 $${\text{Accuracy}} = \frac{TP + TN}{{TP + TN + FP + FN}}$$

7 $${\text{F1-score}} = \frac{2TP}{{2TP + FP + FN}}$$

Accuracy is defined as the proportion of all samples that have been successfully classified. Recall rate measures the proportion of actual positive results that are correctly identified. Specificity indicates the proportion of actual negatives that are correctly identified. Precision is the ratio of samples correctly classified as positive to all classified samples. F1-score is the harmonic average of accuracy and sensitivity. When the above performance index is larger, the classification performance is better.

To verify the effectiveness of our proposed method and the superiority of the network, comparative experiments were carried out with Alexnet [22], Vgg-16 [24], GoogLeNet [25], ResNet-50 [26], DensNet-121 [27] and SqueezeNet [28]. According to the correctness of the sample classification results, four values that constitute the confusion matrix could be obtained: TP, TN, FP and FN, which were substituted into formulas (3), (4), (5), (6), (7) to calculate each performance metrics of the model. The experimental results are shown in Table 2.

**Table 2:** Performance comparison of local projection methods in ancestry estimation

Convolutional network	Accuracy (%)	Precision (%)	Recall (%)	F1-score (%)	Specificity (%)	Para size (MB)
AlexNet	90.83	89.38	93.17	91.97	87.46	233
Vgg16	62.17	69.70	57.76	63.23	67.89	528
GoogLenet	95.92	96.11	96.58	96.65	95.01	51
ResNet-50	96.33	96.74	98.09	97.15	95.09	101
DenseNet-121	97.97	97.98	98.15	98.19	98.55	46
SqueezeNet	92.91	93.76	93.58	93.70	90.88	**5**
**ANINet (SE-Wide)**	**98.04**	**98.76**	**98.44**	**98.27**	**99.01**	45

The significance of bold means that in a certain aspect (performance, model size), this model is the best among these models

It can be seen from Table 2 that the accuracy rate of ANINet is 98.04%, the precision rate is 98.76%, the recall rate is 98.44%, the F1-score is 98.27%, the specific value is 99.01%, and the parameter size is 45 M. Among these experimental models, it has the best effect on the estimation of skull ancestors. Because the network uses a combination of ordinary convolution and extended convolution to extract more spatial features of samples, and the special Wide module improves the training speed of the network and the accuracy of the results.

From the perspective of performance indicators, ANINet's classification accuracy, precision, recall rate, F1-Score, and specificity are all higher than the baseline model, AlexNet. This is because AlexNet has a large number of parameters, which is not conducive to training. Compared with SqueezeNet, GoogLenet, ResNet-50, and DenseNet-121, the metric values of ANINet are higher, because ANINet's extended convolution can extract richer skull ancestry features than traditional convolution. Compared with other networks, Vgg-16 has the lowest classification performance. One is because the gradient disappears during the backpropagation of Vgg-16, and the other is because the complexity of Vgg-16 does not match the order of magnitude of the sample data, which causes an overfitting problem happening.

In terms of parameters, SequeezeNet, which has a lightweight design and maintains good accuracy, has the smallest amount of parameters, followed by ANINet, DenseNet, GoogLenet, ResNet-50 and AlexNet, and the Vgg-16 network has the largest amount of parameters. Compared with the Vgg-16 network, ResNet-50 and GoogLenet use the average pool layer to replace the traditional fully connected layer. Such a modification method effectively reduces the number of network parameters. In addition, DenseNet-121 reduces the number of channels for each convolution input and output, so the parameters of the BN layers and the fully connected layers will also be reduced. ANINet reduces the scale of traditional networks, so it has fewer network parameters.

### Experimental results of global projection

**Fig. 11:**
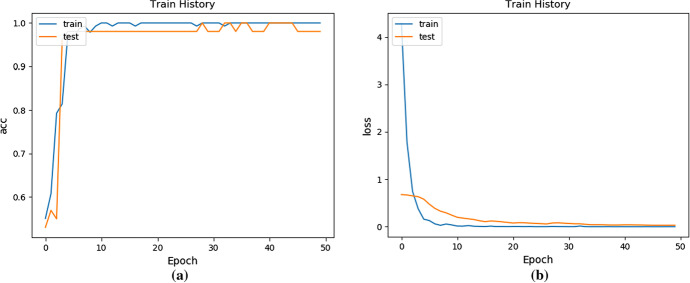
The accuracy and loss of using ANINet change with the number of iterations: **a** The curve of accuracy change; **b** The curve of loss value changer

We used the global projection method to obtain 205 depth images of 629 × 301, reset the image size to 160 × 160 as the input. In the global projection method, we used the fivefold cross-validation method for verification, and the network-related parameter settings are the same as the local projection method. Figure 11 shows the curve of accuracy and loss with the number of iterations.

It can be seen from Fig. 11a that on the training set, the ANINet ancestry estimation accuracy rate can reach 99.37%, and the ANINet ancestry estimation accuracy rate on the test set can reach 98.64%. In the initial training stage, the accuracy of the increase in the number of network training increases. When the number of iterations reaches 40, the accuracy of the network's estimation of skull ancestry reaches the highest value and tends to be stable.

It can be seen from Fig. 11b that on the training set, the lowest ANINet loss value is 0.15, and the lowest ANINet loss value on the test set is 0.13. In the initial training stage, both loss values increase with the number of network training. However, when the number of iteration steps reaches 40, the loss of the network reaches the lowest value and tends to be stable.

To verify the effectiveness of our proposed method and the superiority of the network, Alexnet, Vgg-16, GoogLeNet, ResNet-50, DenseNet-121, and SqueezeNet were selected for comparative experiments. The experimental results are shown in Table 3.

**Table 3:** Performance comparison of global projection methods in ancestry estimation

Convolutional network	Accuracy (%)	Precision (%)	Recall (%)	F1-score (%)	Specificity (%)	Para size (MB)
AlexNet	95.04	92.26	96.27	94.80	92.01	233
Vgg-16	60.78	64.94	57.68	61.17	62.31	528
GoogLenet	96.08	97.31	94.41	95.96	93.69	51
ResNet-50	98.03	98.06	98.38	98.50	97.98	101
DenseNet-121	98.09	98.15	98.43	98.70	98.85	46
SqueezeNet	95.91	96.89	96.47	96.80	93.00	**5**
**ANINet (SE-Wide)**	**98.21**	**98.49**	**98.51**	**98.77**	**99.09**	45

The significance of bold means that in a certain aspect (performance, model size), this model is the best among these models

It can be seen from Table 3 that the five performance metrics of ANINET, which include recall rate, precision, accuracy, specificity and, F1-score, are all superior to those of other neural networks. Compared with other models, the change of ANINET's accuracy index is consistent with the change of many other indexes.

The ANINet network has the best skull ancestry estimation performance, with an accuracy rate of 98.21%; DenseNet-121 and ResNet-50 also have good results, with accuracy rates of 98.09% and 98.03%, respectively; in addition, the accuracy rates of the GoogLeNet, SqueezeNet, and AlexNet networks have reached 96.08%, 95.91%, and 95.04%, while the identification accuracy rate of Vgg-16 is only 60.78%. ANINet has the best ancestry estimation effect mainly for the following reasons: (1) The ANINet network is specifically designed for skull ancestry classification. The neural network is small in size and it is not easy to overfit during training; (2) The Wide module and SE block are used in the network. Feature extraction and feature learning capabilities are strong. The Vgg-16 network, which has a large scale and a large amount of parameters, has a serious overfitting phenomenon during the training process, so the accuracy rate obtained is the lowest. The classification accuracy of the AlexNet network is 3.17% lower than that of ANINet. Because the convolution kernel in the AlexNet network is larger and the number of full connections is large, the network is not easy to train; the estimation accuracy of GoogLenet is 2.13% lower than that of ANINet, because ANINet has added the special SE-Wide module, which adjusts and improves the convolution structure of the traditional network. The classification accuracy of ANINet is 2.40% higher than that of SqueezeNet. Although the amount of network parameters proposed in this paper is higher than that of SqueezeNet, the former's network architecture makes it have stronger feature extraction capabilities. In addition, the performance of ANINET on small-sample data sets such as the skull data sets is slightly better than that of ResNet-50 and DenseNet-121, with an accuracy rate of 0.18% higher than ResNet-50 and an accuracy rate of 0.12% higher than DenseNet-121.

### Experimental results of combining two projection methods

**Fig. 12:**
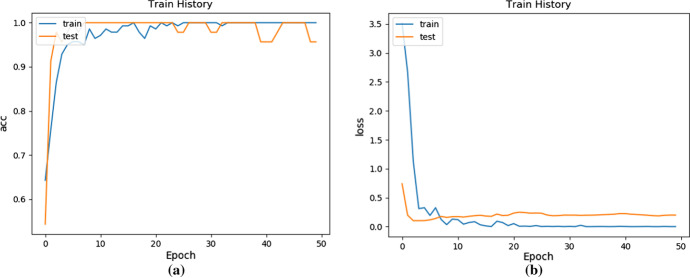
The accuracy and loss of using ANINet change with the number of iterations: **a** The curve of accuracy change; **b** The curve of loss value changer

The depth images obtained by the two projections were fused at the feature channel level, which play a role as input of the network. In the case of combining the two projection methods, we used the fivefold cross-validation method for verification. The number of fully connected layers in the network was set to 1000, and the parameters are the same as the local projection method. Figure 12 is the curve of accuracy and loss value with the number of iterations.

It can be seen from Fig. 12a that on the training set, the ANINet ancestry estimation accuracy rate can reach 99.51%, and the ANINet ancestry estimation accuracy rate on the test set can reach 99.03%. In the initial training stage, the accuracy of the increase in the number of network training increases. When the number of iteration steps reaches 20, the network's accuracy of skull ancestry estimation reaches the highest value.

It can be seen from Fig. 12b that on the training set, the ANINet loss value is the lowest 0.17, and the ANINet loss value on the test set is the lowest 0.11. In the initial training stage, both loss values increase with the number of network training. However, when the number of iteration steps reaches 20, the loss value of the network reaches the lowest value and tends to be stable.

To verify the effectiveness of our proposed method and the superiority of the network, we fused the depth imagess obtained by the two projections and selected Alexnet, Vgg-16, GoogLenet, ResNet-50, DenseNet-121, and SqueezeNet, respectively for comparative experiments. The experimental results are shown in Table 4. The depth images obtained by the two projections were fused at the feature channel level, and the data was divided into training set and test set according to 7:3. The fivefold cross-validation method was used for verification, and the accuracy rate was 99.03%.

**Table 4:** Comparison of accuracy of projection methods

Internet	AlexNet (%)	Vgg-16 (%)	GoogLenet (%)	Resnet-50 (%)	DenseNet-121 (%)	SqueezeNet (%)	ANINet (%)
*Local projection*							
Accuracy	90.83	62.17	95.92	96.33	97.97	92.91	**98.04**
Precision	89.38	69.70	96.11	96.74	97.98	93.76	**98.76**
Recall	93.17	57.76	96.58	98.09	98.15	93.58	**98.44**
F1-score	91.97	63.23	96.65	97.15	98.19	93.70	**98.27**
Specificity	87.46	67.89	95.01	95.09	98.55	90.88	**99.01**
*Global projection*							
Accuracy	95.04	60.78	96.08	98.03	98.09	95.91	**98.21**
Precision	92.26	64.94	97.31	98.06	98.15	96.89	**98.49**
Recall	96.27	57.68	94.41	98.38	98.43	96.47	**98.51**
F1-score	94.80	61.17	95.96	98.50	98.70	96.80	**98.77**
Specificity	92.01	62.31	93.69	97.98	98.85	93.00	**99.09**
*Combining two projection methods*							
Accuracy	95.56	65.00	98.22	98.30	98.36	96.28	**99.03**
Precision	92.97	69.92	97.32	98.49	98.57	97.04	**98.82**
Recall	96.83	58.26	96.97	98.51	98.77	96.87	**98.86**
F1-score	94.93	64.21	98.03	98.74	98.85	97.84	**98.93**
Specificity	94.15	68.88	96.33	98.79	98.99	94.69	**99.11**

The significance of bold means that in a certain aspect (performance, model size), this model is the best among these models

It can be seen from Table 4 that, compared with a single local projection method or a global projection method, the local + global projection method has a good effect in the four networks. The reason is that the combination of the two methods can provide richer features for the network. The local depth projection is used to obtain the independent depth characteristics of the front, side, and bottom parts, and the global depth projection is used to obtain the depth characteristics of the skull. Combine the two to make the characteristics of the input network more diversified.

It should be noted that the classification effect of Vgg-16 is indeed better than that of AlexNet on a large data set, but it is verified in this paper that the classification effect of AlexNet is better than that of Vgg-16 by using our experimental dataset, which is a small-scale dataset. This is because VGG-16 has a large amount of parameters and high space complexity, which does not match the order of magnitude of the training set used in this article, and there is an over-fitting problem at the same time. We compared Vgg-16 with AlexNet in the experiment of local projection, experiment of global projection and experiment of combing two projection methods. The experiment showed that all indicators of Vgg-16 are inferior to AlexNet.

In order to verify the reliability of the above experiments, we introduced a symmetric confidence interval based on the t distribution to measure the variance of the metric value. The confidence of the confidence interval indicates the degree of confidence in the interval estimation, and the interval length of the confidence interval can measure the accuracy of the confidence interval. Weighing the two factors of confidence and interval length can accurately measure the performance of the confidence interval. Generally, the symmetric confidence interval format of t distribution with confidence is as follows:

8 $$\left[ {\hat{\mu } - c\sqrt {\hat{\sigma }^{2} } ,\hat{\mu } + c\sqrt {\hat{\sigma }^{2} } } \right]$$

where $\widehat{\upmu }$ is the sample mean, ${\widehat{\updelta }}^{2}$ is the sample variance, and c is the quantile of the t distribution with K-1 degrees of freedom. The accuracy measurement value based on K-fold cross-validation is the statistical quantity of the sample, so the confidence interval can be rewritten as:

9 $$\left[ {Accuracy - c\sqrt {\hat{\sigma }_{Accuracy}^{2} } ,Accuracy + c\sqrt {\hat{\sigma }_{Accuracy}^{2} } } \right]$$

where

10 $$\hat{\sigma }_{Accuracy} = \frac{1}{K(K - 1)}\sum\nolimits_{k = 1}^{K} {(Accuracy_{k} - Accuracy)}^{2}$$

This paper compares the confidence and interval length of multiple classifiers with the skull experiment data set obtained by local + global projection. The classifiers are Alexnet [22], Vgg-16 [24] and GoogLenet [25], ResNet-50 [26], DenseNet-121 [27], SqueezeNet [28], ANINet (method in this paper). All experiments were repeated 1000 times to avoid the influence of random selection of training set and test set. Choose five-fold cross-validation, K = 5, and the experimental results are shown in Table 5.

**Table 5:** Confidence coefficient (1 − α) and confidence interval (CI) for accuracy based on different classifier models

	AlexNet	Vgg16	GoogLenet	Resnet-50	DenseNet-121	SqueezeNet	ANINet
1 − α	88.7	90.5	95.2	94.6	98.4	95.6	99.1
CI	0.956 (0.929, 0.982)	0.650 (0.443, 0.857)	0.982 (0.973, 0.991)	0.983 (0.966, 0.999)	0.984 (0.977, 0.992)	0.963 (0.957, 0.962)	0.990 (0.987, 0.993)

As can be seen in Table 5, the confidence levels of AlexNet, Vgg-16 and ResNet-50 are all lower than 95%, while the confidence levels of SqueezeNet, DenseNet-121 and the ANINet designed in this paper are all higher than 95%. A 95% confidence level indicates that the confidence interval contains a 95% probability that the true mean of the population is included.

Under an acceptable degree of confidence (95%), the confidence and interval length of the network ANINet proposed in this paper are 99.10% and 0.006. When the confidence level is higher than that of other networks, the interval length of the confidence interval of the network in this paper is reduced by nearly half or even more than half comparing with that of the confidence interval of other networks, which indicates that ANINet is more reliable and credible than other networks in the work of skull ancestry identification.

### Validation of network

To verify the effectiveness of the Wide and SE modules added in this article, we design four different skull ancestry estimation networks, namely, the ANINet network (AlexNet) without the Wide module, and the SE module, and the ANINet network with a single SE module (SE-AlexNet), the ANINet network (Wide-AlexNet) that joins a single Wide module, and the ANINet network (ANINet) that joins the Wide module and the SE module at the same time, and use local, global, local + global three methods to perform different skull depth images recognition. In the experiment, we used five-fold cross-validation to record the recognition accuracy and network parameters of the four network structures in three different ways. The results are shown in Table 6.

**Table 6:** Effects of different strategies on the results

	Convolutional network	Accuracy (%)		Para size	
Local depth images	AlexNet	90.83	− 7.21	45 MB	− 4 k
	SE-AlexNet (SE block)	92.83	− 5.21	45 MB	–
	Wide-AlexNet(Wide)	96.58	− 1.46	45 MB	-4 k
	**ANINet**(**SE-Wide**)	**98.04**	**–**	**45 MB**	**–**
Global depth image	AlexNet	95.04	− 3.17	45 MB	− 4 k
	SE-AlexNet(SE block)	95.93	− 2.28	45 MB	–
	Wide-AlexNet(Wide)	97.47	− 0.74	45 MB	− 4 k
	**ANINet**(**SE-Wide**)	**98.21**	**–**	**45 MB**	**–**
Local + global depth image	AlexNet	95.56	− 3.47	45 MB	− 4 k
	SE-AlexNet(SE block)	97.91	− 1.12	45 MB	–
	Wide-AlexNet (Wide)	98.43	− 0.60	45 MB	− 4 k
	**ANINet (SE-Wide)**	**99.03**	**–**	**45 MB**	**–**

The significance of bold means that in a certain aspect (performance, model size), this model is the best among these models

It can be seen from Table 6 that ANINet (the method in this paper) has the highest recognition accuracy, Wide-AlexNet has the second highest recognition accuracy, SE-AlexNet has the second highest recognition accuracy, and AlexNet has the lowest recognition accuracy. In addition, in terms of parameters, when the SE block is increased, the parameter is only increased by about 4 k. This is because the parameter of SE-block mainly depends on the number of input feature channels, and the number of network feature channels designed in this paper is all a bit less.

As shown in Table 6, compared with the traditional AlexNet network, the SE-AlexNet network has a higher recognition accuracy. This is because the SE block is used to weight the extracted features, which will increase the weight of the features for ethnic estimation, making the extracted features stronger; compared with the traditional AlexNet network, the Wide-AlexNet network has a higher recognition accuracy. This is because the Wide-AlexNet network can obtain richer features, and the wide module in the network uses hole convolution performs feature extraction, so that the feature relationship of nonadjacent spaces in the image will not be interfered by intermediate information, and then a more complete feature space can be obtained; the wide-AlexNet ancestry estimation effect is better than SE-AlexNet, because the Wide module is used as a feature in the main part of extraction, and SE block is mainly further optimize the features extracted by Wide.

ANINet has the best accuracy for skull gender recognition. When we remove SE-Wide and use 1 × 1 convolution combined with ordinary 3 × 3 convolution to perform experiments, the accuracy rate drops. This is because the wide module uses hollow convolution extraction. The feature relationship of the nonadjacent space in the image is not interfered by the intermediate information, and a richer feature is obtained.

### Comparation with state-of-the-art

In order to verify the advancement and effectiveness of the method in this paper, we have selected the four state-of-the-art skull ancestry estimation methods for comparison, namely two-way multivariate analysis of variance [29], geometric morphology analysis [30], GoogLenet [31] and an improved CNN [32]. Herrera and Tallman [29] measured 28 skull index values with ethnic differences, and used two-way multivariate analysis of variance to classify the skull to be tested; Musilová et al. [30] used coherence point shift dense correspondence analysis (CPD-DCA) to register skull data, obtained the coordinate data of the skull vertices through through high-dimensional principal component analysis (PCA) to obtain the skull vertex coordinate data and the obtained principal component scores and ancestry information are used in support vector product (SVM) analysis; Bewes et al. [31] used GoogLenet as the ancestry estimation network framework, which needs 224 × 224 pixel skull computed tomography image as the network input; Wen et al. [26] improved the LeNet5 neural network and input the 6-angle skull images into the improved network, which has an excellent effect on ethnic classification. Therefore, choosing these four methods to compare with the method in this paper has a good representativeness and can also verify the effectiveness of the method in this paper.

The experimental environment of the method in this article and the comparison method were both verified on a computer with NVIDIA GeForce RTX 2080Ti GPU and 28 GB CPU memory. The method and the comparison method in this paper were all used the experimental data introduced in “Experimental data” section to conduct experiments. The accuracy results of different methods of skull ancestry estimation are shown in Table 7.

**Table 7:** The impact of different strategies on results

Methods	Layers	HAN (HA) classification (%)	Uygur (UG) classification (%)	Total classification (%)
Michelle D et al	–	73.68	72.73	73.17
Barbora et al	–	90.85	90.73	90.79
James Bewes et al	22	94.09	93.97	94.03
Yang Wen et al	7	95.54	95.50	95.52
ANINet	5	99.10	98.96	99.03

It can be seen from the above table that ANINet (the method of this paper) has the best estimation effect and the smallest number of network layers. The estimation accuracy rates of Han and Uyghur nationalities have reached 99.10% and 98.96%, respectively. The method of Michelle D et al. has the worst recognition accuracy, only 73.17%. Because the method of Michelle D et al. is a combination of measurement method and multiple linear regression method, there are errors existing in the measurement process, and indicators with ethnic differences are selected randomly, which affects the recognition results; multiple linear regression method is a linear fitting method, and skull features with ethnic differences are a non-linear relationship, and multi-linear discrimination cannot fit non-linear data well. Compared with the method of Michelle D et al., the method of Barbora et al. has a better estimation of skull race, reaching 90.79%. This is because the SVM in the method has a good learning ability for nonlinear classification, but it is also good for reducing the number of hidden variables after the dimension cannot be well estimated. The GoogLenet network used by James Bewes et al. and the improved CNN used by Yang Wen et al. have strong non-linear fitting capabilities. The ancestry estimation accuracy rates reached 94.03% and 95.52%, respectively, and the method requires skull images as data. input, reducing the preprocessing time, avoiding data loss caused by dimensionality reduction or measuring data in the preprocessing, and the training efficiency and prediction accuracy of the model have been greatly improved. The method in this paper also belongs to deep learning and has the advantages of the above methods. In addition, this paper uses a combination of ordinary convolution and extended convolution to extract more spatial features, and adds a unique Wide module to improve the training speed and accuracy of the network. The method in this paper has fewer network layers and higher accuracy. Therefore, compared with other methods, the network effect of this paper is the best.

## Conclusion

To overcome the subjectivity of manual calibration of feature points and improve the accuracy and efficiency of recognition, this paper took the skull depth image as input, combined the Wide module and SE Block to improve the classic AlexNet, designed and proposed an ANINet network. The network used the powerful feature extraction capabilities of the Wide module to combine the SE module and the Wide module to further learn the dependency of feature channels. We obtained the local depth image and global depth image of the skull through depth projection, and then used the global, local, and local + global three methods to experiment on the skull data set and perform cross-validation. The recognition accuracy of the three methods was, respectively, 98.21%, 98.04%and 99.03%. In addition, this paper also designed four different network structures to verify the effectiveness of ANINet. Compared with Alexnet, Wide AlexNet, and SE-AlexNet networks, ANINet has higher accuracy and fewer parameters. Besides, compared with state-of-the art methods, ANINet has fewer network layers, more efficient and accurate expression of data sets, and better recognition capabilities. Therefore, ANINet is an effective method of skull ancestry estimation.

In the study of skull ancestry estimation, this paper first proposed the use of new deep learning technology for skull ancestry estimation, and the efficiency and accuracy have been greatly improved. In the future, our research will focus on improving the accuracy of ancestry classification. We will consider using transfer learning to reduce the cost of model training, so that the model can better adapt to the small dataset; in addition, we are going to use data enhancement methods to improve the classification accuracy. This technology will provide a more reliable reference for practical applications in forensic anthropology, criminal investigation and other fields.

## Data Availability

The datasets analysed during the current study are available from the Northwest University Visual Technology Institute via. The experimental datasets can be submitted to a commitment agreement by email, which shows that the data can only be used for academic purposes, not for commercial purposes. The specific contact person is Professor Geng Guohua, head of Institute of Visualization Technology via, Northwest University, and his email address is ghgeng@nwu.edu.cn.
